# Spatial Patterns of Gallbladder and Biliary Tract Carcinoma in China From 1990 to 2019: An Analysis at the Provincial Level

**DOI:** 10.7759/cureus.42796

**Published:** 2023-08-01

**Authors:** Chuanbo Xie, Di Shi, Hualiang Lin, Yuying Liu, Wei Liu, Peng Yin

**Affiliations:** 1 Cancer Prevention Center, Sun Yat-sen University Cancer Center, Guangzhou, CHN; 2 Department of Epidemiology, Sun Yat-sen University, Guangzhou, CHN; 3 National Center for Chronic and Noncommunicable Disease Control and Prevention, Chinese Center for Disease Control and Prevention, Beijing, CHN

**Keywords:** public health, biliary tract malignancy, disability-adjusted life-years, epidemiology, disease burden, gallbladder carcinoma

## Abstract

Background

Despite a high rate of fatal malignancy, little is known regarding the spatial and temporal patterns of the disease burden of gallbladder and biliary tract carcinoma in China, especially at the provincial level.

Methodology

Using data from the 2019 Global Burden of Diseases, Injuries, and Risk Factors Study, we estimated the temporal trend of the disease burden of gallbladder and biliary tract carcinoma from 1990 to 2019 as well as its incidence, mortality, prevalence, and disability-adjusted life-years (DALYs). We estimated the spatial pattern of the disease burden of gallbladder and biliary tract carcinoma at the provincial level.

Results

The disease burden of gallbladder and biliary tract carcinoma significantly increased from 1990 to 2019 in China. The age-standardized incidence, prevalence, mortality, and DALYs increased by 28.3%, 50.5%, 13.0%, and 7.0%, respectively. The disease burden of gallbladder carcinoma revealed substantial heterogeneity at the provincial level with a higher disease burden in developed provinces or cities than in developing provinces. The disease burden of gallbladder and biliary tract carcinoma was also heavier among males than among females (e.g., age-standardized DALYs: 42.60 per 100,000 people among males vs. 33.57 per 100,000 people among females in 2019).

Conclusions

The disease burden of gallbladder and biliary tract carcinoma has shown rapid changes during the past three decades, with a higher burden in economically advantaged regions than in disadvantaged regions in China. New public health policies and initiatives are needed to address this rising disease burden.

## Introduction

Gallbladder and biliary tract carcinoma is a highly fatal malignancy that may arise from the gallbladder or from the biliary tract. Per the 2018 Global Cancer Observatory (GLOBOCAN), the annual incidence of gallbladder cancer is 2.9 per 100,000 people, accounting for 1.5% of the overall cancer burden worldwide [1]. It is also one of the few cancers that are generally more common among females than among males [2].

During the past several decades, the incidence of gallbladder and biliary tract carcinoma has decreased worldwide, and its associated five-year survival rate has improved due to advancements in chemotherapy and surgical treatment [3]. However, the global incidence and mortality of gallbladder and biliary tract carcinoma have presented striking geographic variations, with a high incidence in Bolivia, Thailand, the Republic of Korea, Chile, and Nepal and a low incidence in European countries [4]. These geographic variations have been attributed to genetic predisposition and differential exposure to some important risk factors, such as obesity and gallstones [5].

China is the world’s largest developing country and has experienced dramatic changes in dietary patterns and lifestyles in recent decades, especially a change from a grain-based diet to an energy-dense and meat-based diet [6]. These dietary changes may substantially affect the incidence of gallbladder carcinoma. However, few studies have systematically investigated the disease burden of gallbladder and biliary tract carcinoma in China. Moreover, given the large geographical regions and specific regional dietary patterns in China, it is critical to understand the spatial pattern of the disease burden of gallbladder carcinoma in China.

The Global Burden of Diseases, Injuries, and Risk Factors Study (GBD) continuously collected estimates of the incidence, prevalence, mortality, and disability-adjusted life-years (DALYs) [7,8]. In 2016, the Chinese Center for Disease Control and Prevention (CCDCP) joined the GBD and provided the incidence, mortality, prevalence, and DALYs of gallbladder and biliary tract carcinoma at the national and provincial levels. In this study, we aimed to systematically examine the spatial and temporal patterns of the incidence, mortality, prevalence, and DALYs of gallbladder and biliary tract carcinoma in China using 2019 GBD data.

## Materials and methods

We used data from the 2019 GBD. The detailed methodology of data collection in the GBD has been reported elsewhere [9-14]. Briefly, the 2019 GBD estimated the burden of gallbladder and biliary tract carcinoma in China using the incidence, prevalence, and mortality, years of life lost (YLLs), years lived with disability (YLDs), and DALYs. The incidence and prevalence data were derived from multiple sources, including national population-representative surveys, the cause-of-death reporting system of the CCDCP, cancer registries, hospital inpatient and outpatient data, and a systematic literature review [15]. The mortality data in mainland China were mainly derived from the National Mortality Surveillance System, Vital Registration, the Notifiable Infectious Disease Reporting System from 2014 to 2015, and Maternal and Child Surveillance System data. The mortality data of Hong Kong and Macao were collected from local medical death certificates.

Standard GBD data processing procedures and analytical models were applied to generate the burden estimates. The descriptions of the standard GBD data analyses have been previously reported [16]. Briefly, we used the Bayesian meta-regression method, i.e., DisMod-MR 2.1, to estimate both the incidence and prevalence of nonfatal outcomes, which were derived from the abovementioned multiple sources. A compartmental model structure with a series of equations was used in the meta-analyses by synthesizing heterogeneous and sparse data. In addition, the Cause of Death Ensemble model (CODEm) platform, a Bayesian, hierarchical, ensemble model designed to estimate cause-specific mortality by year, area, sex, and age, was used to estimate the mortality of gallbladder carcinoma. In the 2019 GBD, all the data sources were adjusted before modeling using the abovementioned standardized approaches.

We used incidence, prevalence, mortality, and DALYs to represent the disease burden of gallbladder carcinoma. Incidence represents the frequency of the disease, which was defined as the number of newly identified cases per 100,000 people. Prevalence represents the magnitude of existing gallbladder and biliary tract carcinoma cases, which was defined as the number of existing gallbladder and biliary tract carcinoma cases (prevalent cases) per 100,000 people. DALYs were used to express the loss of health caused by both the nonfatal and fatal disease burden. DALYs were defined as the sum of YLLs and YLDs. All these metrics were separately estimated for males and females from 1990 to 2019. We defined the age groups in five-year intervals from one year to ≥80 years of age and explored whether the DALYs differed across age groups. Twenty-three provinces, two special administrative regions, four municipalities, and five autonomous regions formed the provincial-level analytic units in this study.

The temporal trends of the incidence, prevalence, mortality, and DALYs of gallbladder and biliary tract carcinoma were analyzed using line charts. The spatial patterns of these metrics across provinces or cities were analyzed using a statistical map graph. Change rates, calculated as the absolute number difference of each metric between 1990 and 2019 divided by the number in 2019, were used to analyze the changes in the disease burden from 1990 to 2019. We further evaluated the annual percentage change of each metric using the Joinpoint Regression Program (version 4.9.1.0, the National Cancer Institute of the United States).

We further explored the associations between the sociodemographic index (SDI) and the age-standardized incidence rates and DALYs using the 2019 SDI and incidence and DALY data at the provincial level. The SDI is an indicator of the development status of each province or city in China based on a province or city’s per-capita income, average years of education, and fertility rate. We first used scatter plots to visualize the associations between the SDI and age-standardized incidence rates and DALYs and then fitted smooth curves between the SDI and the age-standardized incidence rates, the SDI and age-standardized DALYs using a loess method.

The point estimates of incidence, prevalence, mortality, and DALYs were calculated from the mean of 1,000 draw values of each model from the posterior distributions separately by year, province, sex, and age, and the corresponding 95% uncertainty intervals (95% UIs) were calculated with the 2.5th (the lower bound) and the 97.5th percentiles (the upper bound). Data analyses were performed by SAS 9.3 (SAS Institute, Cary, NC) and R software (version 4.2.1).

This study was approved by the Ethics Committee of the National Center for Chronic and Noncommunicable Disease Control and Prevention of the CCDCP. Informed consent was not needed as this study did not include identifiable information about the participants.

## Results

Incidence of gallbladder and biliary tract carcinoma

The number of all-age incident cases and the age-standardized incidence rates in 1990 and 2019 in each province or city in China can be found in Table 1. There were 38,634 (95% UI = 27,351-46,512) incident cases of gallbladder and biliary tract carcinoma in 2019 in China, which was more than three times that in 1990 (12,436, 95% UI = 10,310-20,007). The age-standardized incidence rate increased from 0.37 per 100,000 people in 1990 to 0.48 per 100,000 people in 2019 in China. Increases in the age-standardized incidence rate were seen in all provinces or cities except Hong Kong and Macao. In 2019, Shanghai had the highest age-standardized incidence rate for gallbladder and biliary tract carcinoma (1.07 per 100,000 people), followed by Beijing (0.83 per 100,000 people), Tianjin (0.83 per 100,000 people), and Zhejiang (0.83 per 100,000 people). Qinghai (89.44%) and Shaanxi (83.01%) experienced the highest incidence increases from 1990 to 2019. The age-standardized incidence rate among females was higher than that among males from 1990 to 2012, whereas it was comparable between females and males from 2012 to 2019.

**Table 1:** The numbers of all-age incident cases and the age-standardized incidence rates of gallbladder and biliary tract carcinoma from 1990 to 2019 by province or city in China.

Provinces/Cities	Incident cases, in thousands (95% UI)		Change, %	Age-standardized rate of incidence (95% UI) per 100,000 persons		Change, %
	1990	2019		1990	2019	
China (Total)	12,436 (10,310, 20,007)	38,634 (27,351, 46,512)	210.66 (47.02, 322.34)	0.37 (0.60, 0.31)	0.48 (0.58, 0.34)	28.31 (-38.64, 72.43)
Anhui	612 (480, 1,024)	2,017 (1,176, 2,696)	229.51 (20.74, 403.03)	0.39 (0.63, 0.31)	0.54 (0.72, 0.32)	38.93 (-48.94, 110.78)
Beijing	227 (177, 267)	1,127 (305, 1,492)	396.48 (65.84, 598.11)	0.63 (0.74, 0.48)	0.83 (1.10, 0.23)	31.13 (-55.52, 81.60)
Chongqing	137 (94, 333)	498 (349, 924)	264.14 (125.30, 439.70)	0.29 (0.69, 0.20)	0.28 (0.53, 0.20)	-1.88 (-39.65, 44.09)
Fujian	279 (211, 512)	884 (575, 1,134)	216.87 (22.69, 381.09)	0.35 (0.64, 0.27)	0.43 (0.55, 0.28)	22.01 (-52.42, 83.38)
Gansu	147 (108, 313)	475 (375, 638)	223.67 (69.30, 375.60)	0.27 (0.58, 0.20)	0.33 (0.45, 0.26)	22.04 (-34.45, 76.81)
Guangdong	686 (528, 1,141)	1,863 (1,315, 2,420)	171.47 (25.83, 303.55)	0.37 (0.62, 0.28)	0.34 (0.45, 0.24)	-7.19 (-56.31, 35.21)
Guangxi	344 (259, 660)	613 (435, 1,146)	78.29 (25.32, 152.51)	0.30 (0.57, 0.22)	0.24 (0.46, 0.17)	-17.64 (-41.20, 16.09)
Guizhou	229 (164, 544)	489 (329, 923)	113.76 (39.53, 226.60)	0.27 (0.63, 0.20)	0.27 (0.52, 0.18)	0.30 (-32.96, 50.86)
Hainan	57 (42, 97)	172 (130, 225)	200.23 (64.84, 366.37)	0.31 (0.52, 0.23)	0.37 (0.49, 0.28)	20.50 (-33.14, 84.29)
Hebei	517 (383, 1,002)	1,160 (838, 2,072)	124.29 (60.60, 216.19)	0.29 (0.56, 0.22)	0.29 (0.51, 0.21)	-0.49 (-27.27, 37.98)
Heilongjiang	334 (252, 572)	935 (730, 1,245)	179.69 (71.08, 309.48)	0.41 (0.70, 0.31)	0.39 (0.52, 0.31)	-4.76 (-41.41, 35.40)
Henan	718 (554, 1,236)	2,158 (1,622, 2,763)	200.74 (48.57, 340.16)	0.29 (0.50, 0.22)	0.43 (0.55, 0.33)	48.01 (-27.16, 113.58)
Hong Kong	174 (78, 196)	273 (151, 377)	56.42 (13.70, 183.09)	0.76 (0.88, 0.35)	0.44 (0.63, 0.25)	-41.85 (-57.87, 4.34)
Hubei	540 (431, 871)	1,342 (1,044, 1,768)	148.44 (41.43, 247.90)	0.35 (0.57, 0.28)	0.37 (0.50, 0.29)	6.34 (-36.62, 48.17)
Hunan	516 (385, 1,100)	1,386 (1,067, 1,932)	168.55 (34.67, 292.16)	0.29 (0.61, 0.21)	0.34 (0.48, 0.26)	19.97 (-39.58, 74.58)
Inner Mongolia	168 (127, 315)	615 (478, 788)	265.67 (77.47, 436.26)	0.33 (0.62, 0.25)	0.44 (0.56, 0.34)	34.08 (-35.43, 94.28)
Jiangsu	928 (761, 1,417)	4,055 (1,786, 5,563)	336.83 (44.52, 561.71)	0.43 (0.65, 0.34)	0.72 (0.99, 0.31)	68.19 (-43.52, 151.59)
Jiangxi	325 (240, 698)	738 (573, 1,011)	126.97 (9.26, 240.82)	0.32 (0.70, 0.24)	0.31 (0.43, 0.24)	-2.93 (-52.74, 43.85)
Jilin	230 (174, 458)	674 (556, 884)	193.75 (45.35, 325.50)	0.36 (0.72, 0.28)	0.39 (0.51, 0.32)	7.66 (-46.21, 55.69)
Liaoning	784 (501, 991)	2,120 (1,020, 2,797)	170.58 (70.85, 284.76)	0.67 (0.84, 0.43)	0.66 (0.87, 0.31)	-1.19 (-37.03, 36.94)
Macao	5 (4, 7)	12 (7, 21)	145.66 (51.12, 309.06)	0.43 (0.59, 0.35)	0.32 (0.57, 0.20)	-26.62 (-54.48, 22.27)
Ningxia	34 (26, 58)	170 (113, 222)	401.89 (115.28, 662.07)	0.35 (0.59, 0.27)	0.56 (0.72, 0.38)	61.15 (-31.70, 141.30)
Qinghai	33 (24, 63)	164 (119, 208)	400.95 (118.37, 678.48)	0.33 (0.67, 0.25)	0.63 (0.79, 0.47)	89.44 (-19.20, 188.15)
Shaanxi	412 (325, 583)	1,744 (750, 2,325)	323.27 (36.30, 541.93)	0.45 (0.64, 0.36)	0.82 (1.09, 0.35)	83.01 (-41.87, 171.86)
Shandong	888 (680, 1,565)	2,981 (1,923, 3,807)	235.74 (34.46, 403.14)	0.35 (0.61, 0.27)	0.48 (0.61, 0.32)	37.49 (-44.23, 103.75)
Shanghai	502 (251, 633)	1,912 (362, 2,506)	280.54 (34.49, 412.09)	0.87(1.09, 0.43)	1.07 (1.43, 0.21)	23.04 (-56.47, 61.91)
Shanxi	261 (195, 490)	841 (632, 1,061)	222.21 (59.32, 374.20)	0.33 (0.62, 0.24)	0.44 (0.55, 0.33)	34.74 (-32.76, 97.75)
Sichuan	1,089 (830, 2087)	2,159 (1,684, 2,931)	98.23 (15.67, 184.88)	0.33 (0.62, 0.25)	0.38 (0.52, 0.29)	13.73 (-32.27, 60.22)
Tianjin	157 (126, 189)	716 (264, 957)	356.96 (76.09, 558.53)	0.55 (0.66, 0.44)	0.83 (1.11, 0.30)	50.68 (-40.06, 113.83)
Tibet	17 (10, 55)	29 (20, 66)	74.36 (4.09, 181.66)	0.27 (0.87, 0.16)	0.26 (0.62, 0.18)	-2.37 (-38.56, 53.42)
Xinjiang	119 (87, 256)	440 (328, 643)	270.52 (121.81, 492.50)	0.33 (0.73, 0.24)	0.44 (0.64, 0.33)	33.65 (-22.26, 107.79)
Yunnan	295 (214, 642)	801 (633, 1,180)	171.64 (56.53, 297.09)	0.28 (0.61, 0.21)	0.35 (0.52, 0.27)	22.33 (-28.22, 75.39)
Zhejiang	673 (527, 837)	3,072 (885, 3,978)	356.16 (31.51, 564.39)	0.49 (0.61, 0.38)	0.83 (1.09, 0.25)	68.33 (-50.45, 141.23)

**Figure 1:**
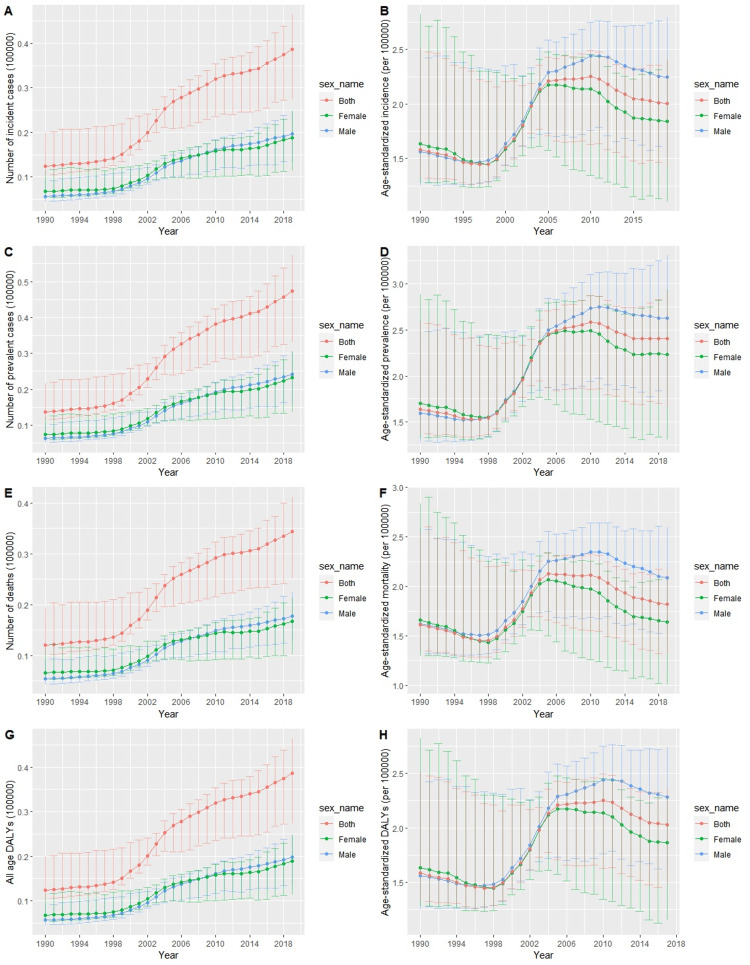
The temporal trends of the numbers of incident cases, prevalent cases, deaths, and DALYs and the age-standardized incidence, prevalence, and mortality rates and DALYs of gallbladder and biliary tract carcinoma in China from 1990 to 2019. DALY = disability-adjusted life-years

**Figure 2:**
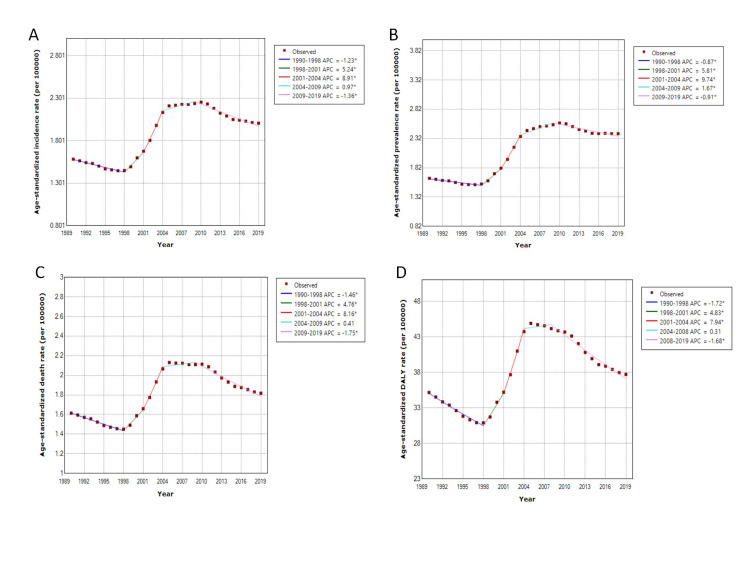
Joinpoint regression analysis for temporal variations in the age-standardized incidence, prevalence, and mortality rates and DALYs of gallbladder and biliary tract carcinoma from 1990 to 2019 in China. DALY = disability-adjusted life-years

**Figure 3:**
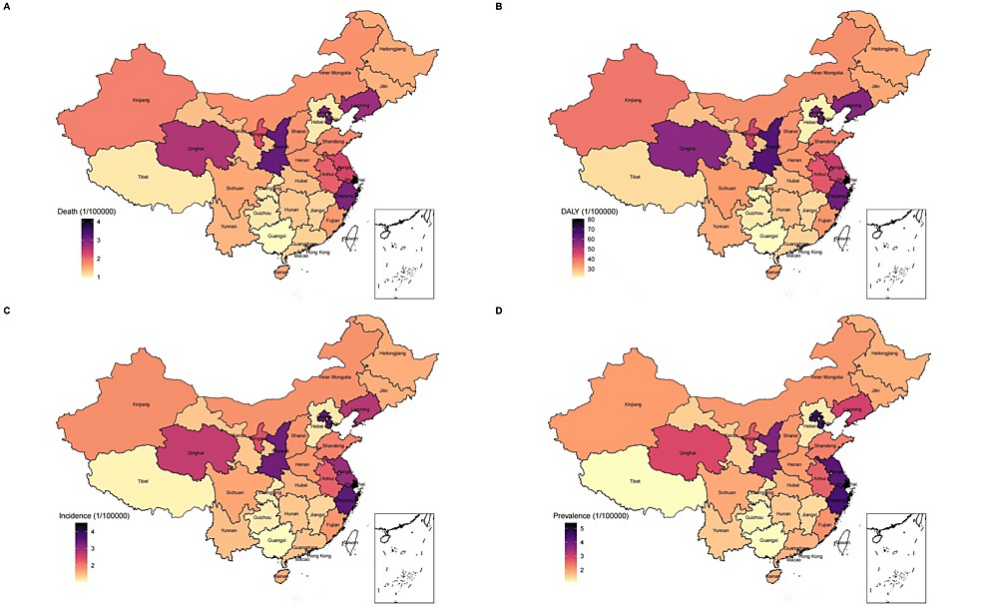
The spatial distributions of the 2019 age-standardized incidence, prevalence, and mortality rates and DALYs of gallbladder and biliary tract carcinoma in China. DALY = disability-adjusted life-years

Figure 1, Panel A, Figure 1, Panel B, and Figure 2, Panel A show the temporal trends of the number of incident cases of gallbladder and biliary tract carcinoma and the age-standardized incidence rates from 1990 to 2019. Although there was a consistent upward trend in the number of incident cases per year from 1990 to 2019, the age-standardized incidence rate decreased from 1990 to 1998, sharply increased from 1998 to 2010, and consistently declined from 2010 to 2019. We also observed that the age-standardized incidence rate among males exceeded that among females after 2004. Figure 3, Panel A shows the 2019 age-standardized incidence rate of gallbladder and biliary tract carcinoma at the provincial level in China.

Prevalence of gallbladder and biliary tract carcinoma

The all-age prevalent cases and the age-standardized prevalence rates in 1990 and 2019 in each province and city in China can be found in Table 2. There were 47,278 (95% UI = 32,889-57,229) prevalent gallbladder and biliary tract carcinoma cases in 2019, which was three times higher than that in 1990 (13,708, 95% UI = 11,345-21,652). The age-standardized prevalence rate of gallbladder and biliary tract carcinoma increased from 1.71 per 100,000 people in 1990 to 2.57 per 100,000 people in 2019. The prevalence of gallbladder and biliary tract carcinoma among females (from 0.78 per 100,000 people in 1990 to 2.56 per 100,000 people in 2019) increased slightly more than that among males (from 0.64 per 100,000 people in 1990 to 2.09 per 100,000 people in 2019). An upward trend in the prevalence was observed in almost all provinces or cities (32 out of 33) from 1990 to 2019 (Table 2). The top three provinces with the highest age-standardized prevalence rate increases were Jiangsu, Zhejiang, and Qinghai. Compared with less developed regions, developed regions such as Shanghai, Beijing, Tianjin, Zhejiang, and Jiangsu had a higher prevalence of gallbladder carcinoma.

**Table 2:** The numbers of all-age prevalent cases and the age-standardized prevalence rates of gallbladder and biliary tract carcinoma from 1990 to 2019 by province or city in China.

Provinces/Cities	Prevalent cases, in thousands (95% UI)		Change, %	Age-standardized rate of prevalence (95% UI) per 100,000 persons		Change, %
	1990	2019		1990	2019	
China (Total)	13,708.42 (11,345.72, 21,652.33)	47,278.77 (32,889.73, 57,229.28)	244.89 (61.48, 370.27)	1.71 (1.42, 2.69)	2.57 (1.77, 3.10)	50.52 (-29.56, 102.96)
Anhui	677.14 (529.63, 1,106.67)	2,380.25 (1,378.83, 3,213.42)	251.51 (29.42, 434.82)	1.73 (1.37, 2.87)	2.79 (1.61, 3.75)	61.26 (-40.34, 144.22)
Beijing	268.64 (207.00, 319.21)	1,506.66 (407.41, 1,999.28)	460.85 (87.02, 697.38)	3.04 (2.35, 3.55)	4.82 (1.30, 6.41)	58.81 (-47.21, 121.56)
Chongqing	149.37 (102.57, 364.26)	588.54 (410.42, 1075.19)	294.01 (143.00, 489.29)	1.30 (0.90, 3.17)	1.49 (1.05, 2.71)	14.73 (-29.38, 69.51)
Fujian	306.08 (230.60, 553.34)	1,073.85 (693.82, 1,386.13)	250.84 (35.24, 437.19)	1.58 (1.20, 2.89)	2.25 (1.47, 2.92)	42.27 (-44.97, 115.45)
Gansu	157.55 (115.94, 332.10)	540.63 (426.43, 729.09)	243.14 (80.98, 407.66)	1.19 (0.88, 2.54)	1.68 (1.33, 2.28)	41.68 (-24.62, 107.33)
Guangdong	763.48 (585.71, 1,254.60)	2,393.66 (1,680.09, 3,097.96)	213.52 (44.98, 369.98)	1.73 (1.33, 2.89)	1.94 (1.36, 2.51)	12.19 (-47.56, 65.74)
Guangxi	361.47 (269.87, 695.70)	711.45 (502.88, 1,316.51)	96.82 (37.82, 178.82)	1.31 (0.98, 2.50)	1.23 (0.87, 2.29)	-5.57 (-32.87, 32.95)
Guizhou	239.18 (170.18, 575.58)	541.25 (362.51, 1,024.50)	126.30 (45.65, 249.24)	1.18 (0.86, 2.79)	1.29 (0.87, 2.44)	9.05 (-28.02, 66.35)
Hainan	61.67 (45.01, 104.12)	201.31 (151.50, 263.28)	226.45 (79.07, 408.07)	1.42 (1.04, 2.41)	1.92 (1.46, 2.51)	35.11 (-25.03, 108.96)
Hebei	559.18 (410.95, 1,098.08)	1,358.39 (983.58, 2,443.52)	142.93 (71.86, 242.28)	1.29 (0.97, 2.50)	1.42 (1.04, 2.53)	9.97 (-20.97, 54.97)
Heilongjiang	379.00 (284.73, 644.88)	1,110.66 (861.75, 1,485.83)	193.05 (79.89, 332.22)	1.85 (1.40, 3.19)	1.96 (1.53, 2.59)	5.70 (-35.43, 51.82)
Henan	769.52 (593.63, 1,337.92)	2,522.74 (1,889.32, 3,243.15)	227.83 (62.16, 380.79)	1.29 (1.00, 2.24)	2.17 (1.63, 2.78)	67.48 (-17.33, 142.10)
Hong Kong	220.77 (99.77, 250.50)	381.49 (210.13, 537.20)	72.80 (23.26, 213.43)	4.19 (1.89, 4.76)	2.90 (1.61, 4.07)	-30.77 (-50.47, 28.38)
Hubei	586.97 (467.69, 948.05)	1,634.24 (1,253.45, 2,150.30)	178.42 (55.98, 294.47)	1.58 (1.26, 2.55)	1.99 (1.54, 2.62)	25.79 (-27.59, 78.26)
Hunan	555.98 (413.59, 1,176.10)	1,612.81 (1,235.16, 2,235.21)	190.08 (44.77, 324.19)	1.27 (0.95, 2.71)	1.74 (1.34, 2.41)	37.04 (-31.58, 99.36)
Inner Mongolia	187.11 (141.41, 346.96)	734.28 (570.48, 940.50)	292.43 (91.41, 480.28)	1.45 (1.10, 2.73)	2.23 (1.74, 2.84)	53.78 (-26.03, 125.58)
Jiangsu	1,082.40 (881.94, 1,618.20)	5,763.64 (2,498.98, 7,973.26)	432.49 (75.20, 707.67)	2.04 (1.66, 3.08)	4.53 (1.98, 6.23)	122.00 (-26.56, 235.28)
Jiangxi	346.80 (254.73, 737.02)	857.73 (665.81, 1,164.30)	147.32 (17.90, 271.15)	1.41 (1.05, 3.03)	1.59 (1.25, 2.17)	12.65 (-45.65, 67.92)
Jilin	260.15 (196.49, 514.65)	819.04 (669.49, 1,079.57)	214.83 (57.29, 360.85)	1.64 (1.26, 3.28)	2.01 (1.65, 2.60)	22.20 (-39.64, 76.86)
Liaoning	829.48 (528.02, 1,059.82)	2,342.72 (1,127.65, 3,098.42)	182.43 (79.27, 305.27)	2.94 (1.91, 3.70)	3.18 (1.53, 4.19)	8.29 (-31.43, 52.82)
Macao	6.02 (4.88, 8.12)	18.19 (11.15, 32.86)	202.00 (83.73, 410.04)	2.32 (1.88, 3.11)	2.13 (1.31, 3.84)	-8.33 (-44.18, 55.17)
Ningxia	37.93 (28.93, 64.43)	204.81 (135.44, 269.70)	439.92 (131.65, 725.08)	1.56 (1.21, 2.69)	2.91 (1.96, 3.79)	86.02 (-20.99, 178.51)
Qinghai	36.83 (27.36, 70.01)	187.87 (135.05, 240.08)	410.05 (125.85, 688.67)	1.50 (1.12, 2.99)	3.05 (2.25, 3.83)	103.66 (-13.17, 212.83)
Shaanxi	476.58 (375.79, 657.52)	2,055.41 (877.44, 2,759.36)	331.28 (40.52, 557.67)	2.15 (1.72, 3.04)	4.13 (1.76, 5.51)	92.30 (-38.68, 190.81)
Shandong	960.15 (735.56, 1,684.75)	3,590.85 (2,329.06, 4,598.72)	273.99 (51.07, 462.97)	1.53 (1.18, 2.70)	2.49 (1.63, 3.19)	62.46 (-33.80, 140.97)
Shanghai	576.73 (286.31, 727.17)	2,303.15 (433.33, 3,035.08)	299.35 (41.91, 442.69)	4.25 (2.12, 5.31)	5.81 (1.10, 7.68)	36.90 (-51.39, 83.83)
Shanxi	285.37 (211.70, 534.50)	1,010.81 (756.57, 1,280.43)	254.21 (76.82, 423.80)	1.45 (1.10, 2.71)	2.26 (1.70, 2.84)	55.36 (-22.69, 127.92)
Sichuan	1,187.11 (900.75, 2,264.66)	2,517.43 (1,954.94, 3,400.89)	112.06 (22.46, 207.93)	1.52 (1.17, 2.87)	2.01 (1.57, 2.72)	32.38 (-22.53, 89.37)
Tianjin	181.61 (144.21, 219.49)	946.15 (347.27, 1,261.61)	420.99 (105.16, 651.35)	2.60 (2.11, 3.12)	4.67 (1.74, 6.19)	79.45 (-28.92, 155.19)
Tibet	17.56 (10.67, 58.53)	32.25 (22.54, 72.80)	83.62 (8.02, 199.64)	1.21 (0.74, 3.99)	1.21 (0.85, 2.77)	-0.05 (-38.91, 58.46)
Xinjiang	133.52 (98.46, 281.09)	504.71 (374.12, 741.91)	278.01 (131.17, 499.64)	1.52 (1.12, 3.32)	2.17 (1.62, 3.16)	42.78 (-16.23, 125.98)
Yunnan	312.58 (223.13, 679.94)	911.99 (715.84, 1,339.55)	191.76 (66.18, 331.80)	1.29 (0.94, 2.80)	1.71 (1.35, 2.50)	32.50 (-23.41, 93.17)
Zhejiang	734.48 (570.84, 914.88)	3,919.83 (1,112.87, 5,073.94)	433.69 (53.38, 677.13)	2.26 (1.77, 2.80)	4.74 (1.36, 6.16)	110.02 (-38.06, 203.27)

Figure 1, Panel C, Figure 1, Panel D, and Figure 2, Panel B show the temporal trends of the prevalent cases and the age-standardized prevalence rates from 1990 to 2019. Similar to the incidence, the absolute number of prevalent cases consistently increased from 1990 to 2019; however, the age-standardized prevalence rates decreased from 1990 to 1998, increased from 1998 to 2010, and then consistently declined from 2010 to 2019. We also observed that the age-standardized prevalence rate among females was higher than that among males during between 1990 and 1998, was comparable between 1998 and 2014, and was finally higher among males than among females after 2004. Figure 3, Panel B shows the spatial pattern of the 2019 age-standardized prevalence rates of gallbladder carcinoma at the provincial level in China.

Mortality of gallbladder and biliary tract carcinoma

Table 3 shows the number of deaths and the age-standardized mortality rates in 1990 and 2019 in each province and city in China. The number of deaths increased from 12,083 (95% UI = 9,983-19,476) in 1990 to 34,462 (95% UI = 25,220-41,231) in 2019. The age-standardized mortality rate increased from 1.61 per 100,000 people in 1990 to 1.82 per 100,000 people in 2019. Most provinces or cities experienced mortality rate increases from 1990 to 2019. The age-standardized mortality rates increased by over 60% for Qinghai and Shaanxi Provinces from 1990 to 2019 but decreased by over 35% in Hong Kong and Macao (i.e., 48% and 38%, respectively).

**Table 3:** The numbers of all-age deaths and the age-standardized mortality rates of gallbladder and biliary tract carcinoma from 1990 to 2019 by province or city in China.

Provinces/Cities	Number of deaths, in thousands (95% UI)		Change, %	Age-standardized rate of mortality (95% UI) per 100,000 persons		Change, %
	1990	2019		1990	2019	
China (Total)	12,083.51 (9,983.25, 19,476.80)	34,462.57 (25,220.93, 41,231.98)	185.20 (35.92, 287.32)	1.61 (1.35, 2.54)	2.08 (1.23, 2.73)	0.13 (-0.45, 0.51)
Anhui	591.86 (460.52, 1,015.25)	1853.84 (1,099.11, 2,446.29)	213.22 (12.43, 373.83)	1.67 (1.34, 2.77)	2.93 (0.83, 3.84)	0.24 (-0.54, 0.84)
Beijing	210.19 (159.44, 249.38)	940.56 (264.02, 1,236.75)	347.48 (49.26, 522.88)	2.57 (1.95, 2.98)	1.82 (1.32, 2.17)	0.14 (-0.60, 0.56)
Chongqing	133.43 (94.36, 315.01)	458.88 (322.36, 865.50)	243.91 (120.95, 412.06)	1.24 (0.89, 2.89)	1.08 (0.76, 2.04)	-0.12 (-0.43, 0.27)
Fujian	272.88 (207.83, 489.11)	795.68 (526.68, 1,005.57)	191.58 (13.15, 331.88)	1.51 (1.17, 2.75)	1.63 (1.10, 2.06)	0.08 (-0.57, 0.58)
Gansu	144.24 (105.58, 309.37)	450.09 (367.95, 609.16)	212.05 (65.34, 349.90)	1.22 (0.91, 2.64)	1.39 (1.13, 1.88)	0.14 (-0.39, 0.60)
Guangdong	659.01 (497.48, 1,103.16)	1,590.49 (1,134.95, 2,043.12)	141.34 (13.19, 247.22)	1.57 (1.20, 2.67)	1.26 (0.91, 1.61)	-0.20 (-0.62, 0.14)
Guangxi	347.50 (262.26, 674.11)	573.99 (404.47, 1,130.83)	65.18 (18.95, 133.29)	1.32 (1.00, 2.55)	0.96 (0.68, 1.91)	-0.27 (-0.47, 0.02)
Guizhou	230.12 (162.41, 543.20)	472.61 (321.47, 899.41)	105.38 (33.69, 218.92)	1.23 (0.89, 2.85)	1.12 (0.76, 2.15)	-0.09 (-0.39, 0.38)
Hainan	56.92 (41.30, 95.44)	161.04 (121.78, 208.24)	182.90 (60.19, 327.54)	1.38 (1.01, 2.30)	1.51 (1.16, 1.94)	0.10 (-0.36, 0.65)
Hebei	511.95 (380.14, 991.86)	1,069.35 (773.42, 1,959.60)	108.88 (53.93, 187.07)	1.24 (0.94, 2.34)	1.11 (0.82, 2.01)	-0.10 (-0.33, 0.22)
Heilongjiang	315.80 (237.48, 532.36)	853.37 (675.67, 1,131.79)	170.23 (75.76, 288.40)	1.72 (1.32, 2.91)	1.51 (1.21, 2.00)	-0.12 (-0.44, 0.22)
Henan	712.74 (562.43, 1,234.15)	1,980.02 (1,517.82, 2,476.33)	177.80 (40.03, 303.94)	1.27 (1.00, 2.14)	1.65 (1.27, 2.04)	0.31 (-0.34, 0.88)
Hong Kong	151.63 (67.74, 170.23)	218.56 (121.67, 300.08)	44.14 (5.11, 157.30)	2.80 (1.25, 3.15)	1.45 (0.81, 2.00)	-0.48 (-0.62,-0.08)
Hubei	527.63 (420.45, 837.56)	1,193.29 (928.12, 1,550.09)	126.16 (35.01, 222.58)	1.52 (1.22, 2.39)	1.42 (1.11, 1.84)	-0.07 (-0.43, 0.30)
Hunan	506.84 (373.06, 1,123.64)	1,286.06 (978.92, 1,810.96)	153.74 (32.69, 280.14)	1.25 (0.93, 2.75)	1.33 (1.02, 1.89)	0.07 (-0.43, 0.57)
Inner Mongolia	161.45 (120.96, 299.64)	557.65 (444.39, 700.46)	245.40 (68.61, 405.25)	1.40 (1.06, 2.57)	1.71 (1.36, 2.14)	0.22 (-0.40, 0.77)
Jiangsu	856.59 (699.23, 1,294.93)	3,104.77 (1,378.05, 4,283.82)	262.46 (21.63, 447.71)	1.73 (1.41, 2.61)	2.29 (1.03, 3.16)	0.32 (-0.56, 0.97)
Jiangxi	319.52 (236.13, 689.50)	688.03 (542.56, 931.49)	115.33 (7.99, 213.52)	1.39 (1.04, 3.00)	1.25 (0.99, 1.71)	-0.10 (-0.54, 0.29)
Jilin	217.64 (165.77, 436.59)	607.98 (494.88, 789.93)	179.35 (38.33, 293.24)	1.53 (1.20, 3.03)	1.47 (1.22, 1.88)	-0.04 (-0.52, 0.32)
Liaoning	793.11 (513.38, 999.91)	2,071.65 (964.25, 2,749.49)	161.21 (67.76, 268.30)	3.00 (1.94, 3.78)	2.75 (1.29, 3.63)	-0.08 (-0.40, 0.27)
Macao	4.43 (3.56, 6.11)	9.19 (5.68, 16.09)	107.52 (27.41, 240.85)	1.63 (1.31, 2.26)	1.01 (0.62, 1.76)	-0.38 (-0.62, 0.02)
Ningxia	32.61 (24.70, 54.38)	154.38 (103.63, 197.09)	373.40 (98.90, 612.73)	1.50 (1.17, 2.54)	2.23 (1.51, 2.80)	0.48 (-0.37, 1.18)
Qinghai	31.35 (23.44, 63.83)	155.46 (114.96, 196.17)	395.96 (115.97, 653.18)	1.46 (1.11, 2.99)	2.60 (1.95, 3.23)	0.79 (-0.23, 1.66)
Shaanxi	392.49 (308.79, 575.83)	1,616.31 (688.14, 2,137.79)	311.81 (29.51, 526.98)	1.93 (1.55, 2.80)	3.18 (1.36, 4.14)	0.65 (-0.47, 1.44)
Shandong	871.94 (659.24, 1540.24)	2,681.65 (1,787.19, 3,388.03)	207.55 (18.49, 360.29)	1.47 (1.12, 2.57)	1.77 (1.19, 2.22)	0.20 (-0.52, 0.77)
Shanghai	475.90 (240.18, 605.97)	1,748.71 (333.76, 2,284.63)	267.45 (31.38, 392.55)	3.63 (1.80, 4.55)	4.16 (0.80, 5.45)	0.14 (-0.59, 0.52)
Shanxi	255.06 (191.38, 487.81)	763.70 (568.12, 966.83)	199.42 (48.82, 344.78)	1.39 (1.06, 2.60)	1.68 (1.27, 2.11)	0.21 (-0.39, 0.76)
Sichuan	1072.76 (834.95, 2004.61)	1,993.32 (1,564.96, 2,723.90)	85.81 (11.15, 162.90)	1.48 (1.15, 2.72)	1.54 (1.21, 2.11)	0.04 (-0.35, 0.45)
Tianjin	146.40 (117.36, 174.91)	596.31 (223.10, 796.28)	307.30 (61.15, 480.30)	2.27 (1.83, 2.65)	2.91 (1.10, 3.85)	0.28 (-0.48, 0.79)
Tibet	16.34 (10.02, 54.49)	28.18 (19.51, 65.79)	72.41 (6.44, 183.21)	1.22 (0.76, 3.99)	1.11 (0.77, 2.61)	-0.09 (-0.41, 0.43)
Xinjiang	113.21 (83.25, 237.72)	416.52 (304.35, 612.20)	267.91 (115.79, 484.51)	1.44 (1.08, 3.03)	1.85 (1.36, 2.69)	0.28 (-0.25, 0.97)
Yunnan	294.07 (214.37, 646.77)	760.86 (600.77, 1,111.58)	158.73 (52.11, 273.42)	1.31 (0.97, 2.84)	1.44 (1.15, 2.14)	0.10 (-0.33, 0.56)
Zhejiang	655.88 (510.21, 815.50)	2,610.07 (804.78, 3,356.29)	297.95 (12.42, 473.10)	2.14 (1.68, 2.62)	2.99 (0.93, 3.85)	0.40 (-0.58, 0.97)

Figure 1, Panel E, Figure 1, Panel F, and Figure 2, Panel C show the temporal trends of the number of deaths and age-standardized mortality rates from 1990 to 2019. The absolute number of deaths consistently increased from 1990 to 2019, but the age-standardized mortality rates decreased from 1990 to 1998, increased from 1998 to 2010, and then consistently declined from 2010 to 2019. The age-standardized mortality rate among females was comparable to that among males from 1990 to 1995, and it was higher among males than among females from 1995 to 2019. Figure 3, Panel C shows the spatial pattern of the 2019 age-standardized mortality rates of gallbladder and biliary tract carcinoma at the provincial level in China.

DALYs of gallbladder and biliary tract carcinoma

Table 4 shows the number of DALYs and the age-standardized DALYs in 1990 and 2019 in each province and city in China. The number of age-standardized DALYs of gallbladder and biliary tract carcinoma increased from 309,016 in 1990 to 763,584 in 2019 and from 35.18 per 100,000 people in 1990 to 37.71 per 100,000 people in 2019, respectively. Qinghai, Shaanxi, and Zhejiang were the top three provinces with the greatest increases in DALYs in China (i.e., over 30%) from 1990 to 2019. In contrast, Hong Kong and Macao had decreased disease burdens of gallbladder and biliary tract carcinoma during the past three decades.

**Table 4:** The numbers of all-age DALYs and the age-standardized DALYs of gallbladder and biliary tract carcinoma from 1990 to 2019 by province or city in China. DALY = disability-adjusted life-years

Provinces/Cities	Number of DALYs, in thousands (95% UI)		Change, %	Age-standardized rate of DALYs (95% UI) per 100,000 persons		Change, %
	1990	2019		1990	2019	
China (Total)	309,016.55 (254,137.86, 486,145.29)	763,584.28 (566,755.02, 920,493.83)	147.10 (19.95, 235.96)	35.18 (29.16, 56.02)	37.71 (27.91, 45.35)	0.07 (-0.48, 0.45)
Anhui	16,265.90 (12,641.83, 26,257.07)	40,041.52 (23,146.28, 52,984.07)	146.17 (-7.74, 280.14)	38.01 (29.74, 62.53)	43.24 (24.90, 57.13)	0.14 (-0.58, 0.73)
Beijing	5,367.11 (3,941.62, 6,557.49)	20,021.81 (5,663.99, 26,632.02)	273.05 (27.10, 427.30)	54.79 (40.44, 65.91)	58.55 (16.62, 77.74)	0.07 (-0.64, 0.48)
Chongqing	3,514.82 (2,479.70, 8,294.27)	9,676.96 (6,750.46, 18,428.60)	175.32 (74.22, 318.42)	27.76 (19.82, 65.18)	22.72 (15.90, 43.02)	-0.18 (-0.49, 0.22)
Fujian	7,064.86 (5,334.75, 12,230.07)	17,967.42 (11,520.47, 22,861.20)	154.32 (-1.61, 283.27)	33.66 (25.51, 58.85)	34.25 (22.27, 43.47)	0.02 (-0.61, 0.52)
Gansu	3,960.13 (2,872.69, 8,236.23)	10,268.93 (8,231.61, 14,051.54)	159.31 (37.54, 282.35)	26.93 (19.87, 57.13)	29.01 (23.40, 39.70)	0.08 (-0.43, 0.56)
Guangdong	16,382.22 (12,234.14, 26,222.68)	36,541.46 (25,459.62, 47,884.56)	123.06 (5.87, 226.35)	34.37 (25.78, 55.86)	26.68 (18.73, 34.88)	-0.22 (-0.64, 0.12)
Guangxi	8,300.07 (6,189.96, 16,174.30)	13,204.23 (9,236.29, 24,764.94)	59.09 (12.80, 126.31)	28.00 (21.02, 54.33)	21.12 (14.81, 39.68)	-0.25 (-0.46, 0.06)
Guizhou	5,873.45 (4,084.75, 14,194.45)	10,591.84 (7,070.35, 19,911.13)	80.33 (14.11, 185.26)	27.08 (19.04, 64.43)	23.57 (15.78, 44.36)	-0.13 (-0.44, 0.37)
Hainan	1,429.06 (1,028.61, 2,343.40)	3,696.05 (2,748.43, 4,826.42)	158.64 (43.20, 301.72)	30.55 (22.17, 50.44)	31.93 (24.01, 41.47)	0.05 (-0.42, 0.60)
Hebei	12,160.10 (8,912.02, 23,925.23)	24,228.67 (17,120.36, 45,110.12)	99.25 (44.25, 176.56)	25.81 (19.01, 50.17)	22.96 (16.36, 42.38)	-0.11 (-0.35, 0.22)
Heilongjiang	8,899.67 (6,580.51, 14,766.44)	20,154.31 (15,660.00, 26,953.34)	126.46 (48.85, 234.49)	38.96 (29.36, 65.50)	31.81 (25.12, 42.05)	-0.18 (-0.47, 0.18)
Henan	17,300.49 (13,635.76, 29,968.04)	43,359.40 (32,803.40, 54,650.48)	150.63 (25.35, 263.24)	26.92 (21.28, 46.49)	33.96 (25.70, 42.50)	0.26 (-0.37, 0.83)
Hong Kong	3,496.42 (1,577.09, 3,923.72)	4,066.84 (2,312.36, 5,690.07)	16.31 (-16.46, 113.96)	60.71 (27.35, 68.06)	28.98 (16.57, 40.64)	-0.52 (-0.66,-0.12)
Hubei	13,287.98 (10,597.83, 21,089.59)	27,194.25 (20,849.54, 35,509.75)	104.65 (17.26, 191.51)	33.07 (26.43, 52.45)	30.30 (23.33, 39.17)	-0.08 (-0.47, 0.30)
Hunan	13,418.99 (9,969.06, 28,877.32)	29,418.06 (22,278.74, 40,016.89)	119.23 (12.60, 225.16)	28.30 (21.00, 61.61)	29.36 (22.33, 39.81)	0.04 (-0.47, 0.54)
Inner Mongolia	4,566.84 (3,406.77, 8,184.56)	13,127.67 (10,268.23, 16,798.52)	187.46 (47.35, 320.31)	31.74 (23.91, 58.17)	35.37 (28.12, 44.77)	0.11 (-0.45, 0.62)
Jiangsu	21,143.09 (17,419.87, 30,208.53)	65,602.59 (28,443.79, 90,347.90)	210.28 (6.50, 361.68)	36.55 (30.03, 53.26)	48.07 (21.02, 65.48)	0.32 (-0.56, 0.96)
Jiangxi	8,697.12 (6,434.88, 18,066.37)	15,560.83 (12,175.11, 20,547.92)	78.92 (-9.72, 164.50)	32.76 (24.30, 69.11)	26.15 (20.56, 34.95)	-0.20 (-0.60, 0.17)
Jilin	6,205.47 (4,693.89, 11,958.35)	14,326.82 (11,270.31, 18,629.97)	130.87 (16.40, 233.66)	35.17 (26.91, 69.71)	31.68 (25.20, 40.94)	-0.10 (-0.56, 0.29)
Liaoning	19,948.30 (12,673.42, 25,177.41)	46,188.84 (21,970.79, 61,670.54)	131.54 (51.35, 234.57)	64.11 (41.30, 80.49)	57.81 (27.55, 76.51)	-0.10 (-0.41, 0.28)
Macao	101.82 (82.66, 135.08)	211.97 (130.24, 378.14)	108.17 (26.05, 249.10)	36.63 (29.66, 48.68)	21.65 (13.36, 38.55)	-0.41 (-0.64, 0.00)
Ningxia	937.23 (708.00, 1,493.52)	3,733.58 (2,530.85, 4,842.26)	298.36 (74.50, 503.01)	34.42 (26.19, 56.57)	47.03 (31.92, 60.34)	0.37 (-0.42, 1.07)
Qinghai	974.78 (722.32, 1,882.54)	3,968.41 (2,886.93, 5,168.68)	307.11 (87.43, 520.96)	35.13 (26.51, 70.48)	56.82 (42.12, 72.65)	0.62 (-0.28, 1.44)
Shaanxi	11,257.90 (8,729.63, 15,050.47)	36,309.84 (15,796.57, 48,144.58)	222.53 (12.49, 395.46)	45.15 (35.47, 62.77)	65.30 (28.36, 86.03)	0.45 (-0.52, 1.20)
Shandong	21,528.30 (16,129.71, 36,933.93)	58,417.80 (39,340.83, 75,209.81)	171.35 (7.65, 303.75)	31.52 (23.82, 54.42)	37.02 (25.06, 47.44)	0.17 (-0.53, 0.73)
Shanghai	11,166.85 (5,453.93, 14,400.70)	35,072.39 (6,621.41, 46,503.05)	214.08 (14.11, 326.30)	75.29 (36.75, 96.34)	80.61 (15.32, 107.06)	0.07 (-0.61, 0.45)
Shanxi	6,623.93 (4,932.02, 12,387.12)	17,578.57 (13,022.41, 22,895.36)	165.38 (34.68, 299.73)	30.60 (23.01, 57.80)	34.78 (25.98, 44.49)	0.14 (-0.43, 0.70)
Sichuan	27,758.50 (21,416.37, 51,310.28)	44,722.57 (34,759.74, 59,741.25)	61.11 (-6.38, 132.73)	32.50 (25.32, 60.08)	33.34 (26.04, 44.24)	0.03 (-0.40, 0.47)
Tianjin	3,620.08 (2,851.05, 4,398.18)	13,494.19 (5,126.03, 17,959.16)	272.76 (49.96, 440.10)	47.28 (38.00, 56.72)	60.25 (22.96, 79.69)	0.27 (-0.48, 0.82)
Tibet	462.50 (283.87, 1,556.34)	765.50 (526.66, 1,754.89)	65.51 (-0.65, 174.42)	29.87 (18.38, 99.19)	25.51 (17.71, 59.41)	-0.15 (-0.47, 0.40)
Xinjiang	3,473.82 (2,590.71, 6,777.20)	10,346.72 (7,450.59, 15,249.00)	197.85 (83.35, 371.48)	35.02 (26.10, 71.49)	39.01 (28.52, 56.97)	0.11 (-0.34, 0.77)
Yunnan	7,734.31 (5,594.68, 16,855.42)	18,100.53 (14,198.28, 25,648.46)	134.03 (35.04, 245.61)	29.79 (21.75, 64.98)	31.14 (24.43, 44.60)	0.05 (-0.39, 0.52)
Zhejiang	16,094.44 (12,548.79, 19,877.47)	55,623.71 (16,854.85, 71,992.52)	245.61 (-1.58, 402.61)	44.96 (35.20, 55.09)	60.86 (18.58, 78.32)	0.35 (-0.61, 0.95)

**Figure 4:**
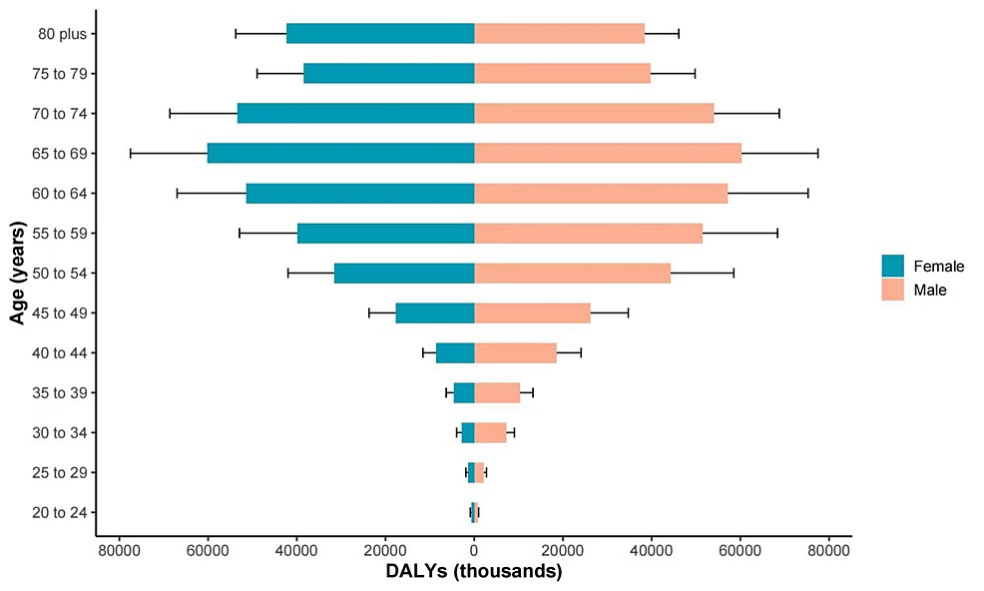
The age-standardized DALYs of gallbladder and biliary tract carcinoma across different age groups in China. DALY = disability-adjusted life-years

Figure 1, Panel G, Figure 1, Panel H, and Figure 2, Panel D show the temporal trends of the number of DALYs and the age-standardized DALYs from 1990 to 2019. Similar to the incidence, prevalence, and mortality, the absolute number of DALYs in China consistently increased from 1990 to 2019. The age-standardized DALYs declined during the period from 1990 to 1998, sharply increased from 1998 to 2010, and then declined again after 2010. Figure 3, Panel D shows the spatial pattern of the 2019 age-standardized DALYs of gallbladder and biliary tract carcinoma at the provincial level in China. Figure 4 shows the distribution of the age-standardized DALYs across different age groups. From Figure 4, we can see that Chinese people aged 65-69 years had the highest gallbladder and biliary tract carcinoma burden among all age groups.

**Figure 5:**
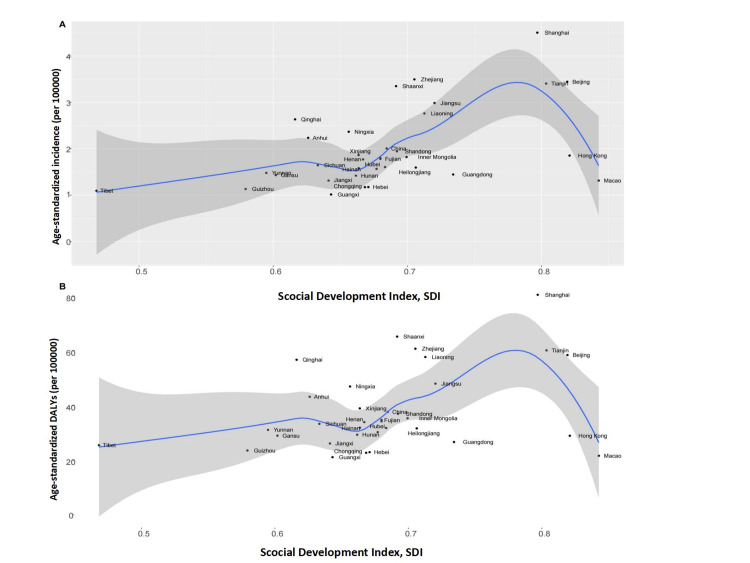
The age-standardized incidence rates and DALYs in different SDI regions in China. The (A) age-standardized incidence rates and the (B) age-standardized DALYs per 100,000 people. DALY = disability-adjusted life-years; SDI = sociodemographic index

Our data also showed a quadratic association between the SDI and age-standardized DALYs. Specifically, when the SDI level was below 0.62 and between 0.67-0.78, the age-standardized DALYs were positively associated with the SDI, and when the SDI level was between 0.62-0.67 and above 0.78, the age-standardized DALYs were negatively associated with the SDI, very similar to the findings for the age-standardized incidence rates (Figure 5).

## Discussion

Using data from the 2019 GBD in China, we found that the age-standardized incidence, prevalence, and mortality rates and DALYs of gallbladder and biliary tract carcinoma sharply increased between 1998 and 2010 and then gradually dropped thereafter. Contrary to the global trend reported by the 2018 GLOBOCAN, our results suggested that the growth rates of age-standardized incidence, mortality, and prevalence rates and DALYs among males were much higher than those among females from 2005 to 2019 in China. The disease burden of gallbladder and biliary tract carcinoma revealed substantial heterogeneity at the provincial level. These findings can be used as a reference for health system decision-making on how to decrease the disease burden of gallbladder and biliary tract carcinoma in China.

Incidence of gallbladder and biliary tract carcinoma

Although the overall incidence of gallbladder and biliary tract carcinoma in China remains at a relatively low level (i.e., lower than 3.0 per 100,000 people), the number and the age-standardized incidence rate of this disease indeed substantially increased from 1990 to 2019. Our findings are similar to the trend observed in a previous study [17]. Improvements in diagnosis (i.e., ultrasound, computed tomography, and magnetic resonance imaging scans) and classification plausibly contributed to this trend. In addition, China is undergoing a dietary pattern transition from a grain-based diet to a high-energy and high-fat Western diet. Such a transition has been associated with increased levels of obesity risk, cholesterol, and bilirubin that collectively could contribute to an increased risk of gallbladder and biliary tract carcinoma [18]. Consistently, we found that the incidence of gallbladder and biliary tract carcinoma was higher in highly urbanized regions, such as Beijing and Shanghai, where high-energy and high-fat Western diets are customary, than in less urbanized regions, where people are more likely to consume traditional grain-based diets. Encouragingly, the incidence of gallbladder and biliary tract carcinoma has slowly decreased during the past 10 years. Although the underlying reasons remain unknown, we suspect that this decline might be largely due to the reduced number of accumulated prevalent cases. We also observed that the decline in incidence varied across provinces, suggesting that health disparities still exist and the need to adopt tailored interventions to ease the regional disparities of the disease burden of gallbladder and biliary tract carcinoma in China.

Prevalence of gallbladder and biliary tract carcinoma

The absolute number of prevalent cases tripled from 1990 to 2019 in China. This can be attributed to an increase in population and improvements in the detection and classification methods for this disease. We also found that economically advantaged regions, such as the Yangtze River Delta, had a higher number of prevalent cases and age-standardized prevalence rates than economically disadvantaged regions. Possible reasons include a larger population size (i.e., over 90% of the participants live in coastal provinces or cities), geographical differences in the incidence of gallbladder carcinoma, and different detection rates across regions in China. Moreover, the prevalence of gallbladder and biliary tract carcinoma still varies greatly at the provincial level. Therefore, more efforts are needed to control the rapid increase in gallbladder and biliary tract carcinoma cases in regions of China where they are highly prevalent, such as the Yangtze River Delta. Our results also support more tailored, province­-specific policies to control the disease burden of gallbladder carcinoma.

Mortality of gallbladder and biliary tract carcinoma

The number of deaths and age-standardized mortality rates of gallbladder and biliary tract carcinoma substantially increased from 1990 to 2019, indicating that gallbladder and biliary tract carcinoma is still a highly fatal malignancy in China. The rise in the number of deaths from gallbladder and biliary tract carcinoma can be attributed to the growing population size (increased from 1.1 billion in 1990 to 1.4 billion in 2017) and the gradually improved vital statistical system over the past decades in China [19]. The overall mortality rate of gallbladder carcinoma in China is similar to the worldwide rate, i.e., 1.82 per 100,000 people in China vs. 1.70 per 100,000 people worldwide [20], but much higher than that in Western countries (e.g., 0.6 per 100,000 people in the United States), suggesting that more efforts are needed to improve the early-stage diagnosis and timely treatment of gallbladder and biliary tract carcinoma in China [21]. Contrary to other countries, such as Bolivia, Thailand, and Chile, where gallbladder and biliary tract carcinoma is more prevalent among females than among males, the sex disparity in the age-standardized mortality rates of gallbladder and biliary tract carcinoma in the Chinese population has reversed, i.e., males have had a higher mortality rate than females since 1994 [22], suggesting that the sex disparity could be more likely driven by environmental and lifestyle factors than biological factors.

Disease burden of gallbladder and biliary tract carcinoma

Using DALYs to quantify the disease burden of gallbladder and biliary tract carcinoma in China, we found that the disease burden of gallbladder and biliary tract carcinoma first increased and then decreased over the past three decades. Compared with Hong Kong and Macao, there was a greater increase in the DALYs in mainland China, possibly due to an increased prevalence of risk factors in mainland China, such as a transition to Western diets. More resources should be invested, especially in regions with high DALYs, to further control the disease burden of gallbladder carcinoma. Controlling lifestyle and metabolic risk factors for gallbladder and biliary tract carcinoma might contribute to reducing its disease burden. Previous studies have reported that <10% of gallbladder and biliary tract carcinoma patients were eligible for surgical treatment at diagnosis, and their mean survival time was only six months [23]. Therefore, early detection is crucial to reduce the burden of gallbladder and biliary tract carcinoma in China. With improvements in diagnostic methods and increasing health awareness in China, the number of early-stage gallbladder and biliary tract carcinoma patients being identified by screening has increased, which might have contributed to the decline in the disease burden since 2010. The Chinese government initiated the Cancer Screening Program in Urban China (CanSPUC) in 2012 [24]. This project provides free screening services for six common cancers, including hepatocellular carcinoma, to urban residents aged 40 to 69 years in 20 provinces. Via this program, residents with a high risk score for hepatocellular carcinoma can also receive a free gallbladder ultrasound examination. In addition to opportunistic screening, these nationwide cancer screening projects in China might also play an additional but critical role in reducing the disease burden of gallbladder carcinoma. Finally, with improvements in adjuvant therapies, especially immunotherapy [25], the five-year survival rate for gallbladder and biliary tract carcinoma patients might improve and help reduce the burden of this disease. Our study also suggested that Chinese individuals aged 65-69 years had the highest DALYs compared to other age groups, highlighting that more medical resources should be allocated to people in this age group.

Despite the interesting insights reported in this study, our study should be interpreted with caution due to several limitations. First, as the data were derived from the GBD, there may have been some inherent limitations [26]. For example, the death distribution methods used to assess the completeness of the Disease Surveillance Point system might have a wide uncertainty range. Second, although the Vital Registration system systematically collects death data in China, data on nonfatal DALYs were insufficient. This might have led to an underestimation of the disease burden for gallbladder carcinoma. Third, the DisMod-MR 2.1 tool did not capture the cohort nature of gallbladder carcinoma.

## Conclusions

The gallbladder and biliary tract carcinoma burden in China has shown rapid changes during the past three decades. The incidence, prevalence, and mortality rates of gallbladder and biliary tract carcinoma declined from 2010 to 2019, which contributed to a consistent decline in its associated DALYs. The disease burden of gallbladder and biliary tract carcinoma varies substantially across different regions in China, with a higher disease burden in developed regions and major cities and a low burden in rural regions. Tailored and targeted interventions should be considered by public health policymakers in China to reduce these disparities.
